# Predictors of COVID-19 vaccine acceptability among health professions students in Vietnam

**DOI:** 10.1186/s12889-022-13236-3

**Published:** 2022-04-28

**Authors:** Cua Ngoc Le, Uyen Thi To Nguyen, Diem Thi Hoang Do

**Affiliations:** 1 Excellent Center for Dengue and Community Public Health, School of Public Health, Walailak University, 222 Thaiburi, Thasala district, Nakon Si Thammarat, 80161 Thailand; 2 Dong Thap Medical College, 312 Nguyen Thai Hoc street, Cao Lanh, Dong Thap Province Vietnam

**Keywords:** Health belief model, COVID-19, predictors of vaccine acceptability, health professions students

## Abstract

**Background:**

The COVID-19 vaccine hesitancy or refusal has actually been a threat to global health. In the current situation, health professions students are at risk for SARS-CoV-2 infection during their internship at healthcare facilities. Furthermore, those future healthcare workers will advise people to accept the COVID-19 vaccination. Therefore, the attitude of students towards vaccine acceptance and the predicting factors needs to be elucidated. This study applied the Health Belief Model to determine predictors of COVID-19 vaccine acceptability among health professions students.

**Methods:**

Nine hundred eleven students participated in a cross-sectional online survey in Vietnam. Data were collected from 1st April to 30th June 2021. Data analysis was performed using SPSS software version 20.0 with Chi-square and Kruskal-Wallis tests before executing multinomial logistic regression to identify predictors of the COVID-19 vaccine acceptability

**Results:**

The overall vaccine acceptance, hesitancy, and refusal rates were 58% (95% CI: 54.7% - 61.3%), 40.4% (95% CI: 37.2% - 43.7%) and 1.5% (95% CI: 0.8% - 2.6%), respectively. Regarding vaccination hesitancy, a predictor such as "Receiving recent flu shots” had a negative correlation, whereas "Vaccines have little efficacy & serious adverse effects” (Perceived barriers), nationality, and majors were positive correlates. For refusal, "Unvaccinated students feasibly infected COVID-19 during hospital internship” (Perceived susceptibility) was a negative correlate. For predicting both hesitancy and refusal, "Mass media appreciating effectiveness and safety of vaccines" (Cues to action), and " Health professions students get serious complications of COVID-19 if not vaccinated" (Perceived severity) were negative predictors. In contrast, "Manufacturers do not disclose adverse effects of vaccines" (Cues to action), and "Adverse effect causes death" (Perceived barrier) were recognized as positive predictors. Strong Health Belief Model predictors of vaccine refusal were "Manufacturers do not disclose adverse effects of vaccines" (Cues to action) with OR= 5.299(95% CI: 1.687-16.641, *p*= 0.004), and "Adverse effect causes death" (Perceived barrier) with OR= 10.255 (95% CI = 3.528-29.814, *p*= 0.0005).

**Conclusion:**

Health professions students' acceptability of COVID-19 vaccination might be based on the perceived susceptibility to and severity of COVID-19, concerns about vaccine efficacy and safety, and the influence levels of information from various sources. Health education and measures to prevent the harmful effects of COVID-19 vaccine misinformation could potentially improve the acceptance rate of the COVID-19 vaccine

## Background

The COVID-19 pandemic has spread worldwide and severely affected the population's health and the national economy [1]. However, the health damage has been mitigated through a series of preventive measures including social distancing, quarantining, face-covering, ventilation of indoor spaces, hygienic behaviors, extensive screening testing, and implementation of government policies such as closing schools and workplaces, banning gatherings in public places, and restricting transportation [2]. Among them, mass vaccination campaigns can be considered an effective way to limit the spread of SARS-CoV-2. Although the COVID-19 vaccines have been available, the success of COVID-19 vaccine coverage depends heavily on the vaccine acceptability of individuals. Achieving herd immunity to significantly prevent the spread of COVID-19 requires a critical immunity threshold of 67% in the general population [3]. However, a recent global survey on COVID-19 vaccine acceptability presented a challenge to achieve this threshold, finding that nearly 30% of participants would refuse or hesitate to use the COVID-19 vaccine [4]. The World Health Organization recommends that strategies are in place to prevent vaccine hesitancy and build confidence in vaccines to develop the maximum effectiveness of available immunization programs [5]. So any vaccination program requires an understanding of the reasons behind COVID-19 vaccine hesitancy as well as strategies to overcome this procrastination [6]. Vaccine refusal or hesitancy has many contributing factors and is present worldwide [7, 8]. A study in the United States showed that access to health information was a positive predictor of vaccine acceptance [9]. Another study conducted among American medical students found that COVID-19 vaccine acceptability was related to concerns about the efficacy and safety of vaccines [10]. The low acceptability of the COVID-19 vaccine use can be influenced by factors such as confidence in the efficacy of the vaccine, fear of side effects [11], and trust levels on the government, public health officials, vaccine developers, and administrators [12]. Lower economic status and lower educational attainment have also been associated with vaccine refusal [13]. In fact, the acceptance of vaccine use has been explained by different health behavior models in which the Health Belief Model (HBM) has been applied to predict preventive health behaviors [14]. Many studies have explored the HBM constructs influencing COVID-19 vaccination, which are crucial for targeted interventions to improve vaccine acceptance [15–18]. According to this model, individuals' beliefs about health and health status play a role in determining health-related behaviors. Five key factors influencing behavior change include: (a) Barriers that may hinder behavior change (Perceived barriers), (b) Benefit to be received from engaging in behavioral changes (Perceived benefits), (c) How susceptibility to illness that each individual think of (perceived susceptibility), (d) What everyone thinks the consequences will be of becoming sick (Perceived severity), and (e) Exposure to information that prompts individuals to act (Cues to actions) [19]. Later, the concept of self-efficacy (Confidence in everyone's ability to succeed) was added to HBM [20]. Self-efficacy is rarely included in studies on the effectiveness of health belief model variables in predicting behavior [21]. Therefore, the original HBM (including 5 key factors) was used in the present study to determine the predictors of acceptability of COVID-19 vaccine use among health professions students. In Vietnam, health professions students were likely to be exposed to COVID-19 patients because they were mobilized to collect clinical specimens for COVID-19 testing due to a lack of health staff [22]. Furthermore, health professions students are future healthcare providers on the front lines of the fight against SARS-CoV-2. They can influence the community through advice and persuasion over vaccines - hesitant people. Moreover, no study to date has explored the acceptability of COVID-19 vaccines among Vietnamese health professions college students. Therefore, this study aimed to assess the acceptability of novel COVID-19 vaccine use as well as to determine predictors among health professions students. The results obtained will identify potential barriers that need to be addressed to ensure adequate vaccine coverage among health professions students and enable the development of health promotion counseling on vaccine-hesitant people.

## Methods

The present study was a cross-sectional online survey conducted among health professions domestic and foreign students in Dong Thap province, Vietnam. In the study, we collaborated with deans and teachers to encourage students to participate in this survey. Study participants who met the following criteria included: (a) health professions undergraduate students; (b) able to access the internet; (c) and submitting informed consent online. All study subjects were informed of the purpose of the study. Anonymity and voluntary participation were guaranteed. The target audience was students of medical laboratory technology, pharmacy, nursing, and physiotherapy who were 18 years old or older.

### Sample size

The sample size was calculated based on the prevalence (p) of 50% (acceptance rate) and using the standard formula of prevalence as follows:

N = z^2^ p (1-p)/ d^2^

At 95% of the confidence interval, Z is 1.96; the acceptance sample error (d) is 5%. The minimum sample size is 400. In this study, the sample size accounted for one-third of the health professions undergraduate population of Dong Thap Medical College, equivalent to 1,000 students chosen from all departments in Dong Thap Medical College, including medical laboratory technology, pharmacy, nursing, and physiotherapy. Since the fourth wave of COVID-19 infections resulted in movement restriction, we implemented a non-probability sampling technique known as convenience sampling. With the support of teachers, questionnaires under Google's form were sent to students through online teaching sessions.

### Measures

The HBM-based structured questionnaire was developed following an extensive literature review on the application of the five HBM constructs in predicting vaccine acceptance and then pretested [15, 16, 23, 24]. It consisted of 4 parts and 26 items. Part I included 6 demographic and health-related items (age, gender, nationality, majors, self-assessment of own health status, and seasonal influenza vaccination). Part II (5 items) focused on the knowledge about name, types, storage, recommended dosage, and adverse effects of the current vaccine used in Vietnam (1 point for 1 item with correct responses). The overall knowledge was categorized by using a cut-off point; good if the score was greater or equal to 60% (≥ 3 points), and poor if the score was less than 60% (< 3 points). Part III (15 items) was designed based on 5 HBMconstructs. (1) Firstly, the perceived susceptibility consisted of 4 items: (a) unvaccinated health professions students have a viable coronavirus infection during their hospital internship; (b) the possibility of spreading COVID-19 to their family and other members of the community from health professions students, (c) the ease with which healthcare workers get COVID-19 from patient care; (d) feasible occurrence of adverse effect of the COVID-19 vaccine. (2) Perceived severity had 2 items: (a) those with chronic illnesses will die without the COVID-19 vaccine; (b) Health professions students develop serious complications from coronavirus infection if they are not vaccinated. (3) The perceived barrier included four items: (a) unsafe vaccines due to rapid research; (b) difficult access to a COVID-19 vaccine if it is not free; (c) fatal vaccine adverse effects; (d) current vaccines have little efficacy and serious adverse effects. (4) The perceived benefits included 2 items: (a) vaccines to prevent the spread of SARS-CoV-2 virus in the community; (b) immunizations protect me from getting coronavirus. (5) Lastly, cues to action had two positive items: (a) health authorities and medical doctors ensure the safety and effectiveness of vaccines; (b) the mass media highly appreciate the effectiveness and safety of vaccines; and a negative item: vaccine manufacturers do not disclose information about adverse effects of vaccines. Each item in HBM constructs was rated using a five-point Likert scale (1 = strongly disagree, 2 = disagree, 3 = neutral, 4 = agree, 5 = strongly agree). Part IV: the last item (outcome variable) was the acceptability of the COVID-19 vaccine use with 3 categories: acceptance, hesitancy, and refusal. In addition, a pilot study was conducted on a sample of 30 health professions students to test the internal reliability of 5 HBM constructs. The content validity of questionnaires of knowledge about COVID-19 vaccines and HBM constructs was assessed by three experts through the Content Validity Index to be 0.93 and 0.95, respectively. Data were collected between 1^st^ April and 30^th^ June 2021 when Vietnam faced a strong fourth wave of COVID-19. Due to the risk of COVID-19 infection, an online questionnaire was designed on Google Forms, a survey management software provided by Google.

### Data analysis

Student feedback was sent to an Excel spreadsheet and then converted to SPSS version 20 data. Before analysis, data was cleaned and outliers were removed. Descriptive statistics were reported for variables related to demography (i.e., age, gender, race/ethnicity, majors), seasonal influenza vaccination, self-perception of own health status, knowledge about current COVID-19 vaccines, HBM factors, and acceptability of COVID-19 vaccine use. This result showed the frequency, percentage of qualitative variables (variables in parts I except age, part III, and part IV), and the mean, and standard deviation of quantitative variables (age, and variables in parts II). Before performing inferential statistics techniques, the Kolmogorov-Smirnov test proved that all continuous variables of HBM constructs were not normally distributed with p < .001. Therefore, a non-parametric test such as Kruskal-Wallis was used to test the differences in the median of HBM constructs scores in three groups of COVID-19 acceptance, hesitancy, and refusal. For the demographic, knowledge, and health-related items in parts I and II, the Chi-square test was applied to determine the relationship with COVID-19 acceptability. Multinomial logistic regression analysis was executed to determine predictors of COVID-19 vaccine acceptability from statistically significant variables in those bivariate analyses. Statistical significance at an alpha (α) level equivalent to .05 was considered for all tests. The outcome variable (Acceptability of COVID-19 vaccination) had 3 categories (acceptance, hesitancy, and refusal) that were not in order. Therefore, multinomial logistic analysis with the stepwise method and forward entry was used to determine predictors of the acceptability of COVID-19 vaccination according to 2 multinomial logit models such as “Hesitancy versus Acceptance” and “Refusal versus Acceptance”.

In order to visualize the COVID-19 vaccination acceptability and measure the performance of classification at various threshold settings, we plotted a ROC (Receiver Operating Characteristic) curve, which showed a plot of 1-specificity (x-axis) versus the sensitivity (y-axis) for a number of different threshold values of predicted probability of the logistic regression model. Because a ROC curve illustrates the diagnostic ability of a binary classifier system; therefore, COVID-19 vaccination acceptability was changed to a binary outcome as "unacceptable vaccination" (True positive) versus “ acceptable vaccination” (False positive). “Unacceptable” implied "hesitancy" or "refusal" against "acceptable". Sensitivity is the probability that the model predicts a positive outcome (unacceptable vaccination) for observation when indeed the outcome is positive. Specificity is the probability that the model predicts a negative outcome (acceptable vaccination) for observation when indeed the outcome is negative. In addition, Area Under the Curve (AUC) was used to assess how well a logistic regression model classifies positive and negative outcomes at all possible thresholds (Predictive performance of a model)

## Results

A total of 911 heath professions students completed the self-administered questionnaires from 1,000 registered students, giving a response rate of 91.1%. The result of the internal reliability of 5 HBM constructs showed Cronbach's alpha to be 0.816. Based on a cut-off point of 0.7 [25], it indicated high internal consistency reliability for HBM constructs scales. As shown in Table 1, the mean age was 20.78 years old and the majority were female (75.4%), pharmaceutical students (62.7%), and Vietnamese students (94.6%). About 50% of students self-rated their health status from good (29.4%) to very good (20.9%). Nearly 60% of students have received a seasonal influenza shot in the past 6 months. In terms of knowledge about the COVID-19 vaccine being used in Vietnam (AstraZeneca), less than 50% of health professions students knew the time interval between 2 doses of vaccine (42.8%) and the dangerous adverse effects (36.5%). In summary, 72.4% of students achieved the level of good knowledge (overall scores ≥ 3 points out of 5 points).

**Table 1:** Health professions student’s characteristics and knowledge about COVID-19 vaccines (*N*=911) (Line 225)

Variables	Frequency (N)	Percentage (%)
**Age**		
19 and less	230	25.7
20 - 21	480	53.6
22 and greater	186	20.8
Mean ± SD = 20.78 ± 1.92		
**Gender**		
Male	224	24.6
Female	687	75.4
**Majors**		
Pharmacy	569	62.7
Nursing	229	25.2
Medical Laboratory Technology	43	4.7
Physiotherapy	67	7.4
**Nationality**		
Vietnamese	862	94.6
Cambodian	8	0.9
Lao	41	4.5
**Seasonal influenza shot in the last 6 months**		
Yes	539	59.4
No	368	40.6
**Self-assessment of own health status**		
Very good	190	20.9
Good	268	29.4
Normal	443	48.6
Bad	7	0.8
Very bad	3	0.3
**Knowledge about COVID-19 vaccine**	**Correct** **N**	**%**
1/Vaccine being currently applied in Vietnam (AstraZeneca)	757	83.1
2/ Category of Astrazeneca vaccine (Viral vector vaccine)	747	82
3/ Storage temperature for AstraZeneca vaccine (From 2^0^ C to 8^0^ C)	650	71.4
4/ Recommended dosage of AstraZeneca vaccine (2 doses with interval of 8 to 12 weeks)	390	42.8
5/ Rare and dangerous adverse effect of AstraZeneca vaccine (Blood clot)	326	36.5
Classification of knowledge		
Poor (overall score < 3)	251	27.6
Good (overall score ≥ 3)	660	72.4

As seen in Table 2, more than 50% of health professions students agreed on beliefs about the susceptibility to COVID-19 infection and vaccine side effects, as well as the benefits of vaccination. More than 40% of health professions students agree with their beliefs about the severity of COVID-19. Agreements on beliefs about barriers ranged from 20% to 40%. In cues to action, about 60% of health professions students agreed on confirmation of the effectiveness and safety of the COVID-19 vaccine from the government and the media. The proportion of health professions students who would accept, hesitate and refuse COVID-19 vaccination was 58.0% (95% CI: 54.7% - 61.3%), 40.4% (95% CI: 37.2% - 43.7%), and 1.5% (95% CI: 0.8% - 2.6%), respectively.

**Table 2:** Levels of HBM constructs and acceptability of COVID-19 vaccine (*N*=911) (Line 234)

HBM constructs	Strongly Disagree (1 point) N (%)	Disagree (2 points) N (%)	Neutral (3 points) N (%)	Agree (4 points) N (%)	Strongly Agree (5 points) N (%)
**Perceived susceptibility**					
1/ Unvaccinated students infected with SARS-CoV-2 during hospital internship	32 (3.5)	20 (2.2)	144 (15.8)	508 (55.8)	206 (22.6)
2/ Possible spread of COVID-19 to family and others from HP students	28 (3.1)	60 (6.6)	195 (21.5)	491 (54.1)	123 (14.7)
3/ The ease with which healthcare workers get COVID-19 from patient care	24 (2.6)	23 (2.5)	147 (16.2)	483 (53.3)	230 (25.4)
4/ Feasible occurrence of adverse effect of the COVID-19 vaccine	29 (3.2)	154 (16.9)	188 (20.7)	467 (51.4)	71 (7.8)
**Mean ± SD:** 15.03 ± 2.54					
**Perceived severity**					
5/ Those with chronic illnesses will die without the COVID-19 vaccine	29 (3.2)	122 (13.5)	282 (31.1)	389 (42.9)	84 (9.3)
6/ Students get serious complications of COVID-19 if not vaccinated	25 (2.8)	51 (5.6)	187 (20.6)	512 (56.5)	131 (14.5)
**Mean ± SD:** 7.15 ± 1.46					
**Perceived barriers**					
7/Vaccine not safe due to rapid research	17 (1.9)	211 (23.2)	420 (46.1)	211 (23.2)	52 (5.7)
8/Difficult access tovaccination if not free	26 (2.9)	171 (18.9)	344 (37.9)	309 (34.1)	57 (6.3)
9/Adverse effects of vaccine cause death	41 (4.5)	216 (23.8)	293 (32.2)	328 36.1)	31 (3.4)
10/Current vaccine having little efficacy and serious adverse effects	39 (4.3)	259 (28.4)	394 (43.2)	190 (20.9)	29 (3.2)
**Mean ± SD:** 12.30 ± 2.64					
**Perceived benefits**					
11/Vaccine effectively prevents the spread of the SARS-CoV-2 virus	41 (4.5)	26 (2.9)	176 (19.4)	528 (58.2)	136 (15)
12/Immunization protecting me from getting coronavirus	25 (2.8)	41 (4.5)	197 (21.7)	499 (55.1)	144 (15.9)
**Mean ± SD:** 7.50 ± 1.46					
**Cues to actions**					
13/ Health authorities ensure safe and effective vaccination	21 (2.3)	30 (3.3)	193 (21.3)	539 (59.4)	124 (13.7)
14/ Mass media appreciate the effectiveness and safety of the Covid-19 vaccine	27 (3.0)	12 (1.3)	149 (16.4)	549 (60.5)	170 (18.7)
15/ Manufacturers do not disclose information about the adverse effects of vaccines	23 (2.5)	171 (18.9)	417 (46)	253 (27.9)	42 (4.6)
**Mean ± SD:** 10.83 ± 1.67					
**Outcome variable: Vaccine acceptability**					
Do you accept to get vaccinated?					
1.Yes	2. Need time to think again			3. No	
Acceptance	Hesitancy			Refusal	
N (%)	N (%)			N (%)	
95% Confidence	95% Confidence Interval			95% Confidence	
Interval (CI)	(CI)			Interval (CI)	
525 (58.0%)	366 (40.4%)			14 (1.5%)	
95% CI: 54.7% - 61.3%	95% CI: 37.2% - 43.7%			95% CI: 0.8% - 2.6%	

In Table 3, the bivariate analysis showed the significant relationship between all the variables in Part I (except gender), II, III, and the acceptability of COVID-19 vaccination (p < 0.05).

**Table 3:** Bivariate analysis of factors associated with Covid-19 vaccine acceptability among health professions students (*N*=911) (Line 236)

Factors	Covid-19 vaccine acceptability			Total	χ^2^
	Acceptance	Hesitancy	Refusal		
Age					
19 and less	135 (58.7%)	87 (37.8%)	8 (3.5%)	230	20.7***
20 – 21	258 (54.2%)	216 (45.4%)	2 (0.4%)	476	
≥ 22	123 (66.8%)	57 (31%)	4 (2.2%)	184	
Gender					
Male	143 (64.1%)	76 (34.1%)	4 (1.8%)	223	4.9
Female	382 (56%)	290 (42.5%)	1 (1.5%)	682	
Majors					
Pharmacy	269 (47.7%)	285 (50.5%)	10(1.8%)	54	71.2***
Nursing	180 (78.9%)	46 (20.2%)	2 (0.9%)	228	
Medical Laboratory Technology	27 (62.8%)	16 (37.2%)	0	43	
Physiotherapy	46 (68.7%)	19 (28.4%)	2 (3%)	67	
Nationality					
Vietnamese	489 (57.1%)	354 (41.4%)	13(1.5%)	856	6.3*
Cambodian	5 (62.5%)	3 (37.5%)	0	8	
Lao	31 (75.6%)	9 (22%)	1 (2.4%)	41	
Seasonal influenza shot in the last 6 months					
Yes	339 (63.2%)	188 (35.1%)	9 (1.7%)	536	14.9**
No	185 (50.7%)	175 (47.9%)	5 (1.4%)	365	
Self-assessment of own health status					
Very good	118 (62.4%)	68 (36%)	3 (1.6%)	189	21.5**
Good	176 (65.9%)	86 (32.2%)	5 (1.9%)	267	
Normal	224 (51%)	209 (47.6%)	6 (1.4%)	439	
Bad	6 (85.7%)	1 (14.3%)	0	7	
Very bad	1 (33.3%)	2 (66.7%)	0	3	
Knowledge about COVID-19 vaccine					
Poor	119 (48.2%)	123 (49.8%)	5 (2%)	247	13.5**
Good	406 (61.7%)	243 (36.9%)	9 (1.4%)	658	
**HBM Constructs**	Mean Rank			χ^2^	
	Acceptance	Hesitancy	Refusal		
**Perceived susceptibility**					
1/ Unvaccinated HP students infected with coronavirus during hospital internship	510.16	379.6	199.93	83.05***	
2/ Possible spread of COVID-19 to family and others from HP students	496.34	392.09	317.57	46.05***	
3/ The ease with which healthcare workers get COVID-19 from patient care	510.04	367.05	428.14	78.29***	
4/ Feasible occurrence of adverse effect of the COVID-19 vaccine	475.51	418.54	444.25	12.11**	
**Perceived severity**					
5/ Those with chronic illnesses will die without the COVID-19 vaccine	470.98	423.78	381.36	9.09*	
6/ HP students get serious complications of COVID-19 if not vaccinated	499.04	388.83	255.46	56.97***	
**Perceived barriers**					
7/Vaccine not safe due to rapid research	405.61	514.18	630.57	49.87***	
8/Difficult to access the COVID-19 vaccine if not free	434.10	470.58	578.43	8.47*	
9/Adverse effects of vaccine cause death	397.39	519.32	732.04	70.07***	
10/Current vaccine havinglittle efficacy and serious adverse effects	387.44	541.87	588.25	89.12***	
**Perceived benefits**					
11/Vaccine effectively prevents the spread of the SARS-CoV-2 virus in the community	497.27	387.18	373.75	50.22***	
12/Immunization protecting me from getting coronavirus	500.37	386.74	260.23	58.72***	
**Cues to actions**					
13/ Health authorities and physicians ensure that everyone is vaccinated safely and effectively	522.50	353.10	325.54	121.43***	
14/ Mass media highly appreciate the effectiveness and safety of the Covid-19 vaccine	504.92	381.85	280.43	70.69***	
15/ Vaccine manufacturers do not disclose information about the adverse effects of vaccines	406.09	507.37	670.14	48.65***	

* *p* < .05; ** *p* < .01; *** *p* < .001

In Table 4, multinomial logistic regression analysis identified predictors of COVID-19 vaccine acceptability among statistically significant variables in the result of bivariate analysis. The multinomial logistic regression model predicted health professions students into one of three categories of COVID-19 vaccine acceptability. By default, SPSS uses the highest-numbered category as the reference category. Therefore, vaccine acceptance with the highest number (525 cases) was used as a reference group [26]. For 2 multinomial logit models, the first for “Vaccine hesitancy” relative to “Vaccine acceptance” included 8 statistically significant predictors (p <.05). The second for “Vaccine refusal” relative to “Vaccine acceptance” consisted of 5 statistically significant predictors (*p*<.05). The regression coefficients of these predictors were statistically different from zero (*p* <.05). In this study, no predictive value was found by the multinomial logistic regression models between COVID-19 vaccine acceptability and some demographic and personal factors such as gender, health status, and knowledge about COVID-19 vaccines (*p* >.05).

**Table 4:** Predictors of COVID-19 vaccine acceptability among HP students by multinominal logistic regression with stepwise method (forward entry) (*N*=911) (Line 249)

Outcome variable	B	Wald	Exp (B)	95% CI for Exp (B)
Vaccine acceptability			**OR**	
**Model 1: Hesitancy**^a^				
Constant	-1.715	3.913		
**Cues to actions**				
-1/Manufacturers not disclosing adverse effect of vaccine	0.330	7.421	1.39**	1.097-1.762
-2/Mass media appreciating effectiveness and safety of vaccines	-0.657	20.372	0.519***	0.390-0.690
**Perceived barriers**				
-3/Vaccines having little efficacy & serious adverse effects	0.671	31.739	1.957***	1.549-2.472
-4/Adverse effect leading to death	0.504	21.708	1.656***	1.339-2.047
**Perceived severity**				
-5/HP students get serious complications of COVID-19 if not vaccinated	-0.294	5.983	0.745*	0.588-0.943
**Demographic variables**				
6/Nationality (Lao: Ref.) Vietnamese	1.653	13.711	5.221***	2.177-12.520
7/Majors (Physiotherapy: Ref.) Pharmacy	0.795	5.585	2.215*	1.145-4.285
8/Seasonal influenza vaccination (No : Ref.)				
Yes	-0.484	8.063	0.616**	0.441-0.861
**Model 2: Refusal**^**a**^				
Constant	-6.607	3.392		
**Cues to actions**				
-1/Manufacturers not disclosing adverse effect of vaccine	1.668	8.157	5.299**	1.687-16.641
-2/Mass media appreciating effectiveness and safety of vaccines	-1.095	8.574	0.335**	0.161-0.696
**Perceived barriers**				
-3/Adverse effect leading to death	2.328	18.277	10.255***	3.528-29.814
**Perceived susceptibility**				
- 4/Unvaccinated HP students feasibly infected COVID-19 during hospital internship	-1.065	8.377	0.345**	0.168-0.709
**Perceived severity**				
-5/ HP students get serious complications of COVID-19 if not vaccinated	-0.837	3.956	0.433*	0.190-0.988
**Pseudo-R Square**				
Cox and Snell’s R^2^		0.327		
Nagelkerke’s R^2^		0.423		
Mac Fadden’s R^2^		0.266		

* for *p*<0.05, ** *p* < 0.01, *** *p*<0.001); a: the reference is “Acceptance” ; Ref.: Reference category

In the first model for “Vaccine hesitancy” versus “Vaccine acceptance”, 2 regression coefficients (B) or log-odds of "Mass media appreciating effectivenessand safety of vaccines", and "Health professions students get serious complications of COVID-19 if not vaccinated" were negative such as -0.657, and - 0.294. If a health professions student increased the agreement of these statements by one point, the multinomial log-odds of choosing "Hesitancy" over "Acceptance" would be expected to decrease by 0.657, and 0.294 units, respectively. For recent seasonal influenza shots (B = - 0.484), heath professions students who recently received a flu shot were less likely to hesitate about COVID-19 vaccination than to accept it. These predicting variables had odds ratio (OR) less than one (OR<1) to be suitable for a negative regression coefficient. For “Mass media appreciating effectiveness and safety of vaccines” with OR = 0.518, health professions students were 0.518 times less likely to choose hesitancy of vaccination than acceptance. Similarly, for the belief of "Health professions students get serious complications of COVID-19 if not vaccinated", it would be 0.745 times less likely to select hesitancy of vaccination than acceptance. Then, health professions students having a recent flu shot were 0.616 times less likely to choose “Hesitancy of COVID-19 vaccination” than students not getting a flu shot. Three remaining variables in the first model, which had positive regression coefficients, were "Manufacturers not disclosing adverse effects of vaccines", "Vaccines having little efficacy & serious adverse effects", and "Adverse effect causing death". If the agreement scales were increased by one point, the multinomial log-odds of selecting "Hesitancy" versus "Acceptance" would be expected to increase by 0.330, 0.671, and 0.504 units, respectively. Interpreting with the odds ratio (OR), if the agreement scales of these perceptions were increased, the probability of hesitancy of vaccination would be 1,390, 1,957, and 1,656 times higher than acceptance of vaccination, respectively. For demographic variables like nationality, and majors, Vietnamese and pharmaceutical students were 5.22 times and 2.21 times more likely to choose "Hesitancy" over "Acceptance" compared to Laos and physiotherapy students, respectively. Students of nursing and medical laboratory technology were not predictive of “Vaccine hesitancy”.

In the second model for “Refusal” versus “Vaccine acceptance”, a cue to actions such as “Manufacturers not disclosing adverse effect of a vaccine” and a perceived barrier like “Adverse effect causing death” with positive log-odds (B) were 5.29 times and 10.25 times more likely to choose “Refusal” than “Acceptance”, respectively. Other predictors with negative log-odds such as "Mass media appreciating effectiveness and safety of vaccines", "Unvaccinated heakth professions students feasibly infected by coronavirus during hospital internship ", and "Health professions students get serious complications of COVID-19 if not vaccinated" were 0.33, 0.34, and 0.43 times less likely to select "Refusal" than "Acceptance", respectively. In general, the rate of “acceptance of the vaccine” among health professions students was determined with the highest correct rate (78.3%) compared with the correct rate of hesitation (65%) and refusal (30.8%).

In summary, the predictors in the "Hesitancy versus Acceptance" and "Refusal versus Acceptance" models of which positive regression coefficient (B) were statistically different from 0 (p < 0.05) included "Manufacturers do not disclose adverse effects of vaccines", "Vaccines have little efficacy & serious adverse effects", "Adverse effects causing death", "Vietnamese nationality", and “Pharmacy major”. In contrast, predictors with a negative regression coefficient (B) included “Mass media appreciate vaccine efficacy and safety”, “Unvaccinated health professions student is likely to contract COVID-19during hospital internship”, “Health professions students will develop serious complications of COVID-19 if not get vaccinated”, and “Currently seasonal flu shot”. Nationalities (Vietnamese versus Laos, OR= 5.221, 95% CI: 2.177-12.520, *p*=0.0005), and majors (Pharmacy versus physiotherapy, OR= 2.215, 95% CI: 1.145-4.285, *p*= 0.018) were the strong predictors of "vaccine hesitancy". Strong HBM predictors of vaccine refusal were "Manufacturers not disclosing adverse effects of vaccines" (OR= 5.299, 95% CI: 1.687-16.641, *p*= 0.004), and "Adverse effect causing death" (OR= 10.255, 95% CI = 3.528-29.814, *p*= 0.0005).

Regarding goodness-of-fit indices to assess the capacity of the logistic regression model, Mac Fadden's R-squared is often chosen. It was 0.226 in a range from 0.2 to 0.4 indicating very good model fit [27].

**Fig. 1:**
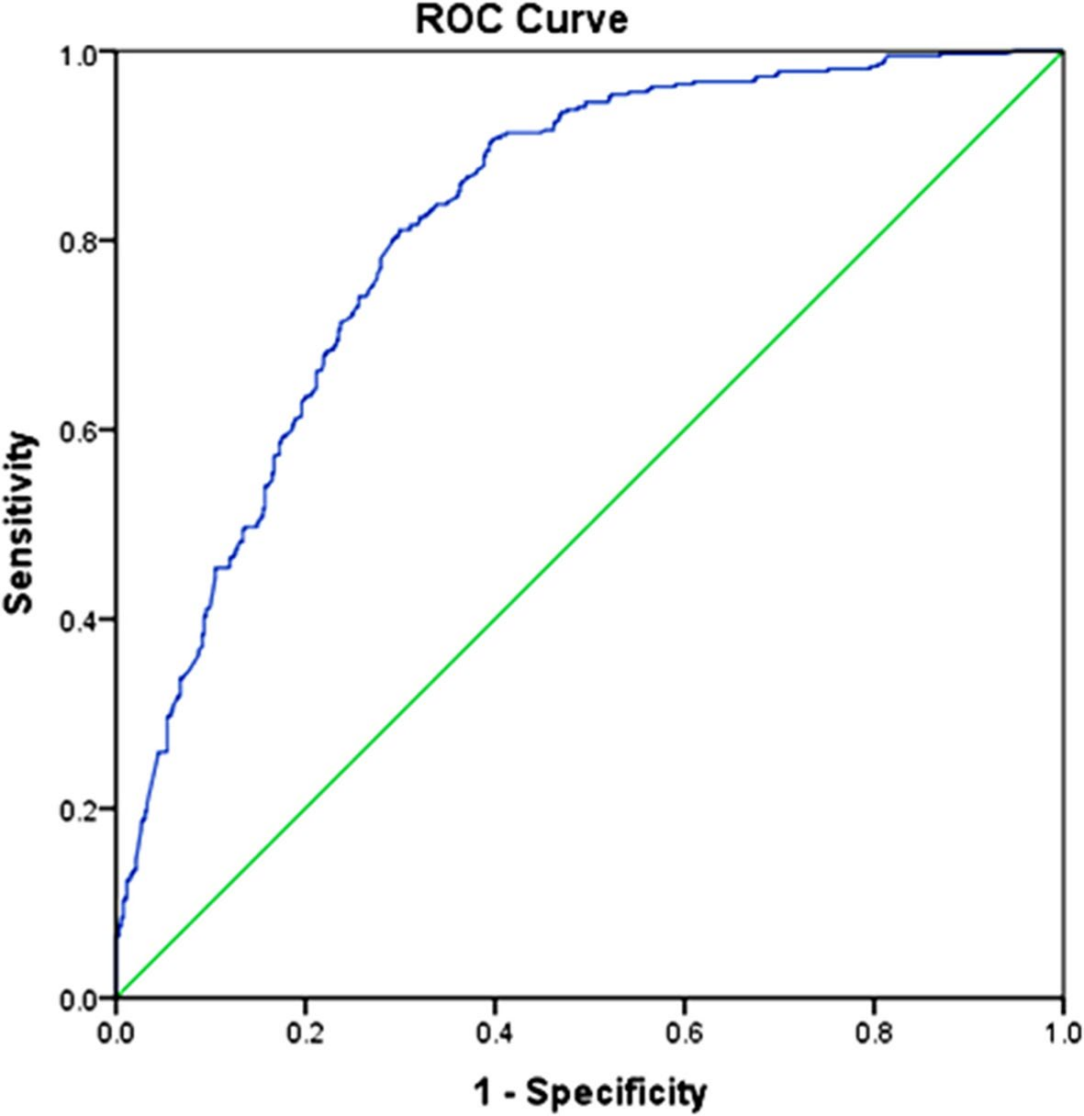
ROC (Receiver Operating Characteristics) curves of the logistic regression model predicting ‘unacceptable vaccination” versus “acceptable vaccination”. (Line 312)

In general, an AUC of 0.5 suggests no discrimination; 0.7 to 0.8 is considered good; 0.8 to 0.9 is considered very good, and more than 0.9 is considered excellent [28]. Table 5 showed an AUC of 0.817 and rejected the test having no discrimination (p<.001). The present logistic regression is a very good model to distinguish between students having an intention of not accepting vaccination and students having intentions of accepting vaccination. This argument is supported by Fig. 1. The ROC curve is close to the top left corner to indicate better performance. Moreover, the curve is far from a 45-degree diagonal of the ROC space to prove the more accuracy of the test [28].

**Table 5:** Areas Under the ROC Curves (AUC) of Logistic Regression (LR) Model predicting COVID-19 vaccination acceptability. (Line 304)

Test Result Variables	AUC	Standard Error	Significance	95% Confidence Interval	
				Lower	Upper
Predicted Probability LR	.817	.014	<.001	.789	.844

## Discussion

The present study explored what the health belief model constructs predicted the acceptability of COVID-19 vaccination among health professions students in Vietnam. Firstly, we found that the proportions of acceptance, hesitancy, and refusal of COVID-19 vaccination were 58%, 40.4%, and 1.5%, respectively. In comparison, the acceptance rate (34.9%) of Egyptian medical students is lower; however, the rate of hesitancy (46%) and refusal (19%) are higher than Vietnamese health professions students [29]. Compared with the results of the study in South Carolina — USA, the acceptance and refusal rates of COVID-19 vaccination among college students were higher than our results with 60.6%, and 24.3%, respectively, except for the rate of hesitancy (15.1%) [30]. In addition to different sociodemographic factors, differences in vaccine acceptability between studies across countries could be traced back to trust in governments and health care systems in each country. The second reason might be a health literacy gap due to insufficient vaccine communication from medical literature and healthcare providers [31]. Communication should deal with anti-vaccine misinformation, and changes in perceptions and behavior. In the current study's cues to action, a predictor in this HBM construct such as "Mass media appreciate the vaccine efficacy and safety" decreased the likelihood for choosing hesitancy and refusal of COVID-19 vaccination by factors 0.519, and 0.335, respectively. In contrast, another predictor such as "Manufacturers do not disclose adverse effects of vaccines" increased the likelihood of hesitant choice and refusal of the COVID-19 vaccine by factors 1.39 and 5,299, respectively. In summary, individuals would be less likely to accept the vaccination if they obtained incomplete information [24]. “Health authorities and physicians ensure that everyone is vaccinated safely and effectively” as the last predictor in cues to action was not associated with the acceptability of the COVID-19 vaccination. In this study, trust in health authorities and healthcare providers was not a driving factor in decision-making among health professions students. The reason might be due to the failure of health authorities and the healthcare system in the control of COVID-19 morbidity and mortality during the 4th wave of COVID-19.The second HBM construct considered as a perceived barrier includes "Vaccines have little efficacy & serious adverse effects", and "Adverse effects cause death" that made health professions students more likely to choose the COVID-19 vaccine hesitancy than acceptance. The results were consistent with the conclusion of a cross-sectional survey on student nurses in the United States. The reasons for the unwillingness to receive the vaccine among student nurses were the belief that the vaccine was developed too quickly to be safe and concerns about the side effects of the vaccine [32]. Vaccine safety concerns due to the development of a new COVID-19 vaccine within a short period have also been mentioned in other studies [33, 34]. Experience from previous influenza pandemics has shown that the use of new vaccines has resulted in low adoption rates in many countries, which promotes a proper understanding of vaccine hesitancy [35, 36]. In the present study, COVID-19 vaccine hesitancy among health professions student is due to the worry about the side effects of AstraZeneca (brain hemorrhage) to occur in healthcare settings during their internship. Teachers and health authorities have not intervened promptly by providing them information with explanations. Due to a lack of official information sources from authorities, misinformation from social media spread and caused skepticism about COVID-19 vaccines. Misinformation became a problem in the outbreak, fueling vaccine hesitancy among a wary public. Such barriers to vaccine acceptance are the product of unfavorable social influences. If most students do not agree on vaccination, they will give a negative signal to others who are likely to accept [37]. Therefore, any effective means of promoting information on vaccine safety also aids in COVID-19 vaccine acceptance. Moreover, if the rush towards early vaccine release is politically motivated and leads to serious adverse effects; the consequence will be the reduction of vaccine acceptance in the public [38]. All findings suggest that health education about the effectiveness and safety of the COVID-19 vaccine in the population is important for the future widespread use of vaccines [39]. In addition, the mainstream press should promote the role of leading public opinion, creating accurate and timely information, helping people use social media to share information from official and reliable sources. Regarding the third HBM construct, the predictor of perceived susceptibility — “Unvaccinated HP students feasibly were infected by SARS-COV-2 during the hospital internship” negatively predicted the refusal of COVID-19 vaccination. This means that health professions students who perceived COVID-19 risk in healthcare practice were more likely to accept COVID-19 vaccination. This is similar to the results of a study conducted in China that also showed that college students who were worried about contracting COVID-19 were more likely to receive a COVID-19 vaccine afterward [40]. In the last HBM construct (Perceived severity), we have also predicted that the explanatory variable “Health professions students get serious complications of COVID-19 if not get vaccinated” would affect the intent to accept vaccination. In summary, we may explain that although students were aware of the importance of the COVID-19 vaccine and accepted the vaccination, they were still hesitant due to uncertainty about the vaccine efficacy and safety.

Regarding sociodemographic characteristics, some variables might predict the acceptability of COVID-19 vaccines. The pharmaceutical students were more likely than physiotherapy students to choose vaccine hesitancy over acceptance (OR=2.215, 95% CI: 1.145 - 4.285). This may be due to different perceptions about the importance of the COVID-19 vaccine in disease prevention. Among health professions students, there were Cambodians and Laotians studying at Dong ThapMedical College. Because the percentage of Cambodians participating in the study was very small (0.9%), the multinomial logit model could only determine Vietnamese students as hesitant to get the COVID-19 vaccine compared to Lao students (OR = 5.221, 95% CI: 2.117 – 12.520). The reason might be the limit of the Vietnamese language. Thus, Lao students were less affected by negative information, anti-vaccine communication, fake news, etc. With respect to previous use of the flu vaccine, health professions students who had used it in the past 6 months were less likely to hesitate about receiving the COVID-19 vaccine. The survey on factors influencing attitudes toward COVID-19 vaccination in the United States also demonstrated that vaccination history was the most important predictor of intention to vaccinate against COVID-19 [32]. Therefore, the prior usage of flu vaccines might reflect the confidence of health professions students in vaccine safety.

### Limitations of study

There are potential limitations to this study. First, self-reports were used to answer the questions. Reporting bias may be a limitation. Subjects can present themselves under favorable conditions. However, ensuring anonymity reduces this bias. Second, due to restrictions on the movement and gathering of people during the COVID-19 outbreak, random sampling cannot be applied. Therefore, the convenience sampling method was adopted in this study, which may lead to selection bias, and poor representativeness. The last, study design is a cross-sectional survey. Therefore, we cannot confirm the causal relationship between predictors and the acceptability of COVID-19 vaccines.

## Conclusion

Health professions students in Vietnam might select one among three choices in the acceptability of COVID-19 vaccination based on the perceived susceptibility to and severity of COVID-19, concerns about vaccine efficacy and safety, and the influence levels of information from various sources. Health education and measures to prevent misinformation could potentially improve the acceptance rate of the COVID-19 vaccine. Regarding preventing misinformation about the COVID-19 vaccine, every country needs to put in place strict sanctions on the production and distribution of fake news. By providing people with accurate and easy-to-understand information, governments need to educate citizens about COVID-19 vaccination, as well as increase confidence in vaccines. Moreover, it is indispensable for the participation of information technology "giants" such as Facebook, Google, LinkedIn, Youtube, etc. in the fight against misinformation. Ultimately, the acceptability of a COVID-19 vaccine is a global issue. In the context of the world of information chaos, close coordination between countries is required to ensure the dissemination of legitimate information and limit the harmful effects of misinformation about COVID-19 vaccines.

## Data Availability

The datasets generated and/or analysed during the current study are not publicly available due to no permission from the Research Committee of Dong Thap Medical College but are available from the corresponding author on reasonable request.

## Abbreviation

HBM: Health Belief Model
